# Informed consent learning: Needs and preferences in medical clerkship environments

**DOI:** 10.1371/journal.pone.0202466

**Published:** 2018-10-03

**Authors:** Tahra AlMahmoud, M. Jawad Hashim, Rabah Almahmoud, Frank Branicki, Margaret Elzubeir

**Affiliations:** 1 Department of Surgery, College of Medicine and Health Sciences, United Arab Emirates University, Al Ain, UAE; 2 Department of Family Medicine, College of Medicine and Health Sciences, United Arab Emirates University, Al Ain, UAE; 3 Department of Clinical Sciences, College of Medicine, University of Sharjah, Sharjah, UAE; 4 Department of Medical Education, College of Medicine and Health Sciences, United Arab Emirates University, Al Ain, UAE; Public Library of Science, UNITED KINGDOM

## Abstract

**Purpose:**

Limited information exists regarding students’ routine educational needs in support of ethics and professionalism practices faced in real clinical practice. As such the authors aimed to explore medical students learning needs and preferences for informed consent and relevant ethical issues in the clerkship environments.

**Materials and methods:**

A cross-sectional study using a self-administered, printed survey distributed to final year clinical clerks.

**Results:**

84% completed the survey. Students indicated the need for more attention to all topics related to informed consent (mean = 7.1 on a scale of 0 to 9; **±**1.2). Most additional instructional attention was requested for topics raised in discussions with patients concerning the risks, benefits and alternatives to recommended treatments (7.3 ±1.4). The cohort expressed the need for education in the care of vulnerable patients (7.2 ±1.2) with a maximum score for the care of abused children. Women perceived greater need for education concerning informed consent than male respondents (*p*>0.05). There were significant differences between students who scored high or low on the item “being treated in professional manner” and “endorsement of educational needs for care of adolescents” (*p* = 0.05).

**Conclusion:**

There was heightened perception among final year medical students of the need for greater attention to be paid to informed consent education.

## Introduction

Ethical and professionalism queries about the examination and treatment of patients have received ample of interest in recent years [1,2], hence, it is essential that medical trainees become sensitive to ethical issues relevant to patient care and exhibit a scope for proper reasoning. Traditionally, the need for informed consent has been practiced in invasive clinical procedures as well as research involving human contribution, but physicians are more expected to obtain informed consent for other forms of treatment or interventions. The need for proper training becomes important from a medico-legal aspect as well as from the prospects of ethical and moral premises [3].

Transformation in clinical practice patterns with diffuse boundaries in patient care and changing communities standards with an emphasis on non-maleficence and autonomy along with the shift of the domination of care from physician to both the patient and doctor are elemental concepts of informed consent and the trademarks of modern medical ethics [4,5]. Hence, it is crucial that formal learning takes place relating to various elements of informed consent with their medico-legal dimensions, and how to obtain informed consent from patients whose decisional capacity is compromised or presents challenges due to language or limited educational barriers.

In clinical environment interns and residents are often involved in obtaining informed consent, sometimes without adequate supervision; furthermore, they may perceive pressure by senior colleagues to obtain written consent despite their lack of knowledge concerning the risks for possible morbidity and mortality [6]. Yet, the curricula of medical schools often provide little formal training such that learning opportunities, with regard to ethical issues including informed consent, are likely to occur through an informal or hidden curriculum. There is though, a growing recognition among medical educators of the adverse effects of the hidden curriculum and a need to promote the development of ethical considerations for medical students [4,7]. Positively, Hafferty and Franks suggested providing students with appropriate circumstances to learn the relevance of medical ethics in their social contexts [4].

## Theoretical perspectives

Recognizing the complexity of the clinical clerkship learning environment, we considered how the cognitive, social and emotional aspects of learning and teaching of ethical principles might be re-conceptualized to move forward with fulfilment of learning needs. We therefore elaborated the overlapping theoretical perspectives of adult learning theory or andragogy [8], reflective practice [9] social cognitivism [10], experiential learning theory [11] and Bourdieu’s social theory of cultural practice [12]. Adult learning theory posits that adult learners prefer to be autonomous and exercise choice to satisfy learning needs, they need to see the relevance of what they are learning, prefer to take responsibility for their learning and are capable of self-direction. The theory also recognizes that while driven by some external motivators learners are more responsive to internal motivators and prefer to apply knowledge rather than be merely told what to do. We further recognized that the professional practice of medical ethics is both a cognitive and reflective activity. Indeed, a significant characteristic of contemporary medical ethics is perhaps its reflective nature. Schon [9] proposes that reflective practice is an essential aspect of what professionals do. Whether we are learners, teachers or researchers, the complexity of the practices we often encounter demand reflection. These skills include making evaluative judgments about effectiveness in meeting goals and actual performance. Assumptions of social cognitivists are that learners learn from interaction with and observations of others (e.g. role models). Students’ interaction with more experienced peers contributes to cognition and skills being stimulated to more advanced levels; practice of skills in a community of practice thus increasing the likelihood of transfer to new and more complex situations. Learners are also goal oriented and direct their behavior accordingly, eventually being able to reflect on and regulate their own learning and behavior [10]. The theory explains how people acquire and maintain particular behavioral patterns. Experiential learning theorists similarly give experience, and reflection on experience, a central role in their theories of human learning and development [11].

Lastly, we adopted Bourdieu’s social theory of cultural practice [12] as it brought to bear considerations of the impact of the cultural context, or what Bourdieu describes as ‘habitus’ on the analysis of the cognitive content of the ethics course. The habitus of individuals and groups is a central concept in Bourdieuan social theory [13]. As such, our focus is on the medical ethics habitus and learning dispositions of senior medical students in clinical contexts. Thus, the way in which students learn and apply ethical principles such as informed consent is played out in a social field constituted by interactions between teachers, students, patients, the culture of the medical school and hospital etc. All are situated in the broader macro level sociocultural fields of Emirati medicine and healthcare systems. Indeed, as Hafferty and Franks [4] remind us, “formal instruction in medical ethics does not take place within a cultural vacuum”, therefore we need to bear in mind that the views of senior UAE medical students’ are influenced by both the formal, and more subtle, hidden curriculum in the medical ethics classroom, hospital or clinic. The hidden curriculum is defined by Hafferty [7] as “a set of influences that function at the level of organizational structure and culture”. Since students have a well-informed sense of the range of potential opportunities their learning environments afford, the focus of this paper is primarily on connecting the formal and informal pedagogy of ethics teaching and students’ formal contribution to the dialogue about how ethical principles can be better taught and learned.

To our knowledge, there has not been a previously published study exploring the views of medical students on the need for training with a focus on practical ethics and professional development topics in the Middle East region. This study documents opinions of graduating medical students regarding topics in need of greater curricular attention in the areas of informed consent, dealing with complex issues surrounding needed training for effective care of vulnerable patients and principles of education related to broader ethical issues. This may help to promote the efforts of medical educators to create valuable opportunities for adoption of ethical considerations that, in turn, may better equip doctors to optimize their professional roles for the population and the nation that they serve.

## Methods

A cross-sectional study was conducted using a printed and self-administered questionnaire among final year medical students in the United Arab Emirates.

### Instrument

Having obtained Institutional Review Board (IRB) approval (Al Ain Medical District Human Research Ethics Committee), a paper survey with an attached cover letter indicating the purpose of the study and guaranteed anonymity was distributed to medical students and administered in English language. Participation in the anonymous survey was considered as implied consent, as is a standard practice for non-clinical questionnaires. The cover letter indicated invitation of the students to participate in this survey and students had the option to not fill out the questionnaires. The survey tool was adapted from the University of New Mexico; (copyright reserved by Laura Roberts, Cynthia Geppert and Teddy Warner) (received approval for it usage through personal communication, see acknowledgment). This survey instrument eliciting views on professionalism and “ethics” education across ten domains is based on the American Board of Internal Medicine definition of professionalism [14–16]. The ten domains include attitudes, goals, learning methods, curricula, knowledge assessment, skills assessment and educational needs concerning informed consent topics, principles, vulnerable populations, relationship and boundaries[14–17]. Addition five queries regarding personal experiences during training and three demographic questions were included. Items in the survey were rated on a nine point Likert-type scale[16,18,19] (i.e. 1 = much less attention; 2 = moderately less; 3 = slightly less; 4 = seldom less; 5 = same; 6 = slightly more; 7 = moderately more; 8 = frequently, 9 = much more for response to questions “how much educational attention should the topic receive level of teaching”. The responses were transformed into a score by obtaining the arithmetic mean of the numeric code (as listed above). Negatively worded items were first reverse coded e.g. 1 was recoded to 9 and 2 was recoded to 8.

This article reports the opinions of graduating medical students as to whether there is a perceived need for education concerning 44 items related to informed consent, broader ethical issues, and complex ethical, social, philosophical, and legal issues surrounding vulnerable patients.

### Subjects

Inclusion criteria for selection of participants included enrolment in the medical program at the UAE University. Subjects were 108 graduating medical students enrolled at the College of Medicine and Health Sciences, United Arab Emirates University (UAEU). The survey was distributed during surgical specialty rotations from August 2009 to February 2013. Respondents had previously completed clinical clerkships in internal medicine, general surgery, family medicine and psychiatry. The survey was distributed to the final year students as our intention was to explore their perceived experiences about the theory and application of principles with regard to ethics in clinical practice, following exposure to the entire medical school curriculum. We assumed professional attitudes and patterns at this stage of education and training were defined to some extent. The survey was conducted for three academic years to increase the sample size as our numbers of clinical clerkship students have been relatively small. All participants were exposed to the same curriculum and in order to reduce bias, the survey was distributed at the end of the surgical rotation for each group and collected immediately following completion.

### Analysis

Post hoc power analysis revealed that with a sample size of 108, there will be a 99% chance (power) to detect a difference of at least 0.5 standard deviation between two subgroups in statistical analysis, assuming a two-tailed significance level of 0.05.

Standard descriptive statistics were used, and correlation coefficients (r) were calculated using the Pearson correlation coefficient. All analyses were carried out using SPSS for Windows version 20.0 (SPSS, Chicago, IL). The unpaired *t* test was used to evaluate the difference between male and female groups. Cronbach’s α was used to assess internal reliability. Gender differences were detected using analysis of variance (ANOVA). A *P* value less than 0.05 was considered statistically significant. Missing data were not imputed.

## Results

### Respondent characteristics

The questionnaire was distributed to 128 (32 male, 96 female) final year medical students, 108 (22% male and 78% female) responded to the survey. The age data were available for 92 students with mean of 24 years (range 23–27 years).

*Ethical conflicts encountered during training* -*details could be accessed*[20]: Students expressed routinely encountering ethical conflicts during training, and perceived that they had acquired a moderate level of ethics training during medical school. They reported that medical education had helped them to deal with ethical dilemmas. Students also disclosed being evaluated in a professional manner by supervising colleagues.

### Educational needs concerning informed consent principles

The survey requested students to rate 9 items describing consent topics. In the range of 1 to 9 (1 =“much less”, 9 = “much more”) trainees indicated that compared with attention given to this topic in current education and training, more attention to all topics was needed (mean 7.1 **±**1.2 range 3.6–9). Most additional attention requested was in the areas of discussing risks, benefits and alternatives to the recommended treatment with patients (mean 7.3 ±1.4), conducting assessments of decision making capacity (mean 7.3 ±1.7), obtaining informed consent or refusal from surrogate decision makers (mean 7.1 ±1.6), and obtaining informed consent from patients who are capable of making decisions (mean 7.0 ±1.7) (Table 1). Additional attention was requested to a lesser extent on topics, including decisions relating to how much clinical information to share with patients (mean 7.0 ±1.5) and when to withhold information from patients (mean 6.7 ±1.9).

**Table 1:** Compared with your training NOW, how much educational attention should the topic of informed consent receive (Mean ±SD).

	Gender		Overall (N = 108)
Chronbach’s α 0.915	Female (N = 84)	Male(N = 24)	Male and Female
1. Deciding how much clinical information to share with patients	7.00±1.53	6.83±1.52	6.96±1.52
2. Deciding when to withhold information from patients	6.82±1.79	6.13±2.09	6.66±1.87
3. Discussing risks, benefits and alternatives to the recommended treatment with patients	7.40±1.47	7.17±1.34	7.34±1.44
4. Obtaining informed consent from patients who are capable of making decisions	7.06±1.70	6.79±1.67	7.00±1.69
5. Obtaining informed consent from patients whose decisional capacity is compromised	7.11±1.66	6.48±1.53	6.97±1.65
6. Obtaining informed consent from non-Arabic speaking patients	7.04±1.60	6.54±1.53	6.93±1.59
7. Obtaining informed refusal from patients who decline recommended treatment	7.22±1.50	6.50±1.79	7.06±1.59
8. Obtaining informed consent or refusal from surrogate decision-makers	7.18±1.54	6.75±1.68	7.08±1.57
9. Conducting assessments of decision- making capacity	7.39±1.55	6.96±2.05	7.29±1.67
Group means	7.19±1.23	6.67±1.21	7.07±1.24

Rated on a scale from 1 ‘‘much less” to 5 ‘‘same” to 9 ‘‘much more” attention needed compared to now.

There was substantial agreement among respondents of both genders resulting in no statistically significant differences. However, more women requested attention to enable decisions as to when to withhold information from patients (mean 6.8 ±1.8 versus mean 6.1±2.1), obtaining informed consent from non-Arabic speaking patients (mean 7.0 ±1.6 versus mean 6.5 ±1.5), and obtaining informed refusal from patients who decline recommended treatment (mean 7.2 ±1.5 versus mean 6.5 ±1.8) (Table 1).

No significant correlations were, however, observed between mean scores on the topic “educational attention, how much should the topic informed consent receive” and experience of (a) encountering ethical conflicts during training (r = 0.003), (b) having positive role models of ethical and professional conduct among superintendent (r = -0.09), (c) being treated in an ethical and professional manner (r = 0.07). Further analysis also revealed no significant mean response differences for educational needs concerning informed consent topics and principles between the students who thought that they were not treated professionally (mean<5.5) and those treated in professional manner (mean score ≥5.5) on the item “being treated in professional manner” (mean 6.9 ±1.5 versus mean 7.1 ±1.1, *p* = 0.38).

### Dealing with complex ethical, social, philosophical, and legal issues

On a scale of 1–9 (1 = “much less” 5 = same and 9 =“much more”) students rated educational attention needed for 25 topics associated with complex ethical, social, philosophical, and legal issues. They were instructed to indicate how much future attention should be paid to teaching the topics compared with their current training.

There was a clear indication of perceived need for more attention to all curricular topics (mean 7.2**±**1.2) (Table 2). Men gave lower ratings to all items compared to women; however, women gave the following factors statistically significantly more weight: truth-telling and honesty (*p* = 0.003), compassion for suffering (*p<*0.001), respect for human dignity (*p* = 0.01), scientific integrity and research (*p* = 0.001), justice (*p* = 0.03), and responsibility to improve the community (*p* = 0.03). There was no correlation between the scoring of the need for attention to be paid to relevant education and the following mean scores: encountering ethical conflicts during training (r = 0.46), having positive role models of ethical and professional behavior among supervising residents and faculty (r = 0.76), nor for being treated in a professional manner (r = 0.73). Additional analysis showed no significant difference in the mean response between the students who thought that they were not treated professionally (mean<5.5) (group 1) and those treated in an professional manner (mean score ≥5.5) (group 2) on the item “During your medical training, how often have you been treated in a professional manner by your supervising residents, faculty, and training institution” (7.2 ±1.4 versus 7.3 ± 1.1 respectively, *p* = 0.79). Additional analyses indicated no significant differences among students who scored high or low on the item “being treated in professional manner” for endorsement of the need for education.

**Table 2:** Compared with your training NOW, how much educational attention should the broader ethics issues receive (Mean ±SD).

Chronbach’s α 0.934	Gender		Overall (N = 108)
	Female (N = 84)	Male(N = 24)	Male and Female
1. Truth-telling and honesty	7.44±1.35*	6.46±1.50	7.22±1.50
2. Compassion for suffering	7.30±1.26*	6.17±1.40	7.05±1.35
3. Respect for human dignity	7.43±1.47*	6.67±1.58	7.26±1.52
4. Scientific integrity and research	7.12±1.46*	5.96±1.60	6.86±1.56
5. Justice	7.42±1.47*	6.67±1.63	7.25±1.54
6. Respecting the law	7.42±1.47	6.88±1.48	7.30±1.48
7. Responsibility to improve community	7.74±1.35*	7.04±1.46	7.58±1.40
8. Non-discrimination	7.37±1.60	6.96±1.63	7.28±1.61
9. Respecting patient autonomy	7.38±1.65	7.08±1.53	7.31±1.62
10. Faithfully serving patient interests	7.43±1.41	6.88±1.48	7.31±1.44
Group means	7.40±1.16	6.68±1.14	7.24±1.19 (4.60–9.00)

Rated on a scale from 1 ‘‘much less” to 5 ‘‘same” to 9 ‘‘much more” attention needed compared to now

*Statistically significant difference between male and female, p<0.05

### Dealing with issues regarding care of vulnerable patients

The survey requested students to rate 10 topics regarding education concerning how to care for vulnerable patients. Participants were instructed to indicate how much educational attention the topics should receive compared with their current training. The items were scaled from 1 = much less to 9 = much more.

On a scale of 1–9 (1 = much less, 5 = same and 9 = much more) students rated educational attention needed for ethical issues surrounding the care of vulnerable populations compared with the attention currently provided (Table 3). While respondents of both genders agreed that all curricular areas needed more attention (mean score 7.2 ±1.2; range 4.6–9.0), women expressed greater need for attention to all items compared to men (mean = 7.4 ±1.2 versus 6.7 ±1.1 female and male respectively) (Table 3). Statistically significant differences between the genders were observed with women requesting more attention to ethical issues surrounding care of children (*p* = 0.03), care of adolescents (*p* = 0.01), care of women (*p* = 0.02), care of men (*p* = 0.001), care of elderly people (*p*<0.001), care of indigenous (local, national) patients (*p* = 0.03), care of patients who abuse substances (*p* = 0.01), care of patients in rural areas (*p* = 0.01), care of the prisoners of war/combatant detainees (*p*<0.001), and care of military or police personnel (*p*<0.001). Further analysis stratifying student responses into two categories: (1) those who scored lower than 5.5 (29 students) on item “being treated in a professional manner” (2) those scoring more than 5.5 (65 students) revealed statistically significant differences in response to the item additional “educational need for care of adolescents” (mean 6.7 ±1.7 versus 7.3 ±1.4, *p* = 0.05) and care of military or police personnel (mean 6.4 ±1.9 versus 7.3 ±1.5, *p* = 0.01) for students with the lower and higher scores respectively.

**Table 3:** Dealing with complex ethical, social, philosophical, and legal issues surrounding the care of vulnerable populations: Compared with your training NOW, how much educational attention should the topic receive? (Mean ±SD).

	Gender		Overall (N = 108)
Chronbach’s α 0.969	Female (N = 84)	Male(N = 24)	Male and female
1. Care of children	6.83±1.54*	6.08±1.35	6.66±1.52
2. Care of adolescents	7.26±1.46*	6.38±1.44	7.06±1.49
3. Care of women	7.00±1.48*	6.21±1.35	6.82±1.48
4. Care of men	6.90±1.59*	5.71±1.55	6.64±1.65
5. Care of elderly people	7.74±1.28*	6.58±1.61	7.48±1.44
6. Care abused children	8.13±1.18	7.50±1.67	7.99±1.32
7. Care in situations of domestic violence	8.01±1.21	7.38±1.66	7.87±1.34
8. Care of patients with HIV	7.82±1.35	7.59±1.79	7.77±1.44
9. Care of people with infectious diseases with public health consequences	8.04±1.17	7.17±1.52	7.84±1.30
10. Care of mentally ill patients	7.70±1.28	7.13±1.90	7.57±1.45
11. Care of terminally ill patients	7.88±1.34	7.33±1.63	7.76±1.42
12. Care of critically ill patients	7.79±1.252	6.92±1.742	7.59±1.414
13. Care of chronically ill patients	7.38±1.43	6.71±1.73	7.23±1.52
14. Care of indigent (local, national) patients	7.25±1.47*	6.25±2.03	7.03±1.66
15. Care of non-Arabic speaking patients	7.26±1.50	6.92±1.61	7.19±1.52
16. Care of suicidal patients	7.82±1.20	7.25±1.65	7.69±1.33
17. Care of pregnant patients	7.06±1.70	7.04±1.43	7.06±1.64
18. Care of patients from other cultures	6.90±1.51	6.46±1.77	6.81±1.57
19. Care of violent patients	7.40±1.38	6.83±1.52	7.28±1.43
20. Care of patients who abuse substances	7.69±1.26*	6.92±1.47	7.52±1.34
21. Care of people at risk for genetic disorders	7.58±1.35	6.75±1.98	7.40±1.54
22. Care of patients in rural areas	7.55±1.44*	6.54±1.98	7.32±1.62
23. Care of the prisoners of war/combatant detainee	7.68±1.46*	6.29±2.10	7.37±1.71
24. Care of military or police personnel	7.33±1.60*	6.00±1.59	7.04±1.69
25. Care of employees	7.04±1.77	6.33±1.52	6.88±1.74
Group means	7.48±1.05	6.77±1.30	7.33±1.14 (3.80–9.00)

Rated on a scale from 1 ‘‘much less” to 5 ‘‘same” to 9 ‘‘much more” attention needed compared to now.

*Statistically significant difference between male and female, *p*<0.05

## Discussion

The survey offers an insight into how medical students in clinical training perceive informed consent learning and teaching. It revealed the perceived needs of medical students for additional medical ethics education on a diverse set of topics, including informed consent, issues surrounding care of vulnerable patients, and principles of education for broader ethics issues. These findings are in congruence with a study conducted by Roberts et al [15], who found that students in training expressed high interest in curricula awareness to improvements in bioethics principles, informed consent and management of special population related topics [15]. Also consonant with previously published findings [21], students expressed the need for education concerning informed consent topics including decisional capacity, and surrogate decision makers. Respondents in the present study endorsed the need for education on broader ethics issues with the highest score associated with responsibility to improve the community and this may be associated with an underlying passion for entering medicine and a sense of social commitment. These latter aspects are often considered humanistic and professional merits, imperative to exemplary patient care and should be advocated in medical school curricula [22] recognizing that fundamentals of medical professionalism are culture responsive [23]. Furthermore, students asked that more attention be paid to obtaining informed consent from patients who are capable of making decisions. Occasionally, a meaningful and ethical informed consent may vary among cultures; studies have revealed that the concept of disease is perceived through social values and power scales in the family based on cultural systems in some societies and common ethical concepts and practices should be considered with reference to social, cultural and religious contexts [1,24–27]. Depicting on categorizations of the ways people of different cultures relate, Hammami et al [28] hypothesized that the procedure of giving and receiving informed consent can be transformed according to whether it is a collectivist, high context communication culture or an individualistic, low context communication culture. Interestingly, in a collectivist Arab society, where it is often assumed individual decision-making is not the norm; extensive information was required to be disclosed in the informed consent process and in contrast to expectations, patients desired more information regarding benefits and post-procedure issues rather than risks and available alternatives [28]. Medical students can be challenged when translating and fitting in with existing policies and practices that are at odds with each other. Desired information detail will inevitably vary from one patient to another. As del Pozo and Fins [29] suggest, informed consent is an area requiring research to clarify what information to be conveyed to individuals and families, through culturally appropriate approaches. In a study carried out in an Indian village, the majority of respondents interviewed could not decide on clinical trial participation before discussing with community members [24]. The ethical principles of western countries require all adults to be the primary decision makers of their participation, which may not be fully applicable in other systems that are culturally and socially different from the western world. Reference to documents concerning informed consent prepared locally might be somewhat different to documents in other countries where the emphasis on patient autonomy may vary. In a cosmopolitan society, yet unique in its cultural values and social system, the concept of disease and therapy is occasionally perceived through social and family values. For example, in the UAE an adult female patient is unlikely to sign for herself for procedures involving reproduction. These might create uncertainties and confusion in the minds of junior trainees, hence endorsing a need for more curricular attention to these issues. As such, there is a call for cross-cultural comparisons of ethical dilemmas related to consent and educational needs for senior medical students. In our opinion, the topic of informed consent needs to receive greater attention and the guidelines should be based on factors such as culture, patient level of education and demographics and these be discussed with students in the formal curriculum.

Although respondents in the present study expressed interest in additional teaching on all the topics, they demonstrated the greatest interest in learning more about the care of the abused child. This may reflect gaps in the child protection system including medical, legal and social services as have been reported by others in the region [30]. In addition, identification, prevention and management strategies are diverse according to cultural attitudes and behaviors [31]. Although it is expected that physicians perform screening for domestic violence in children, provide primary intervention, support, information, protection and reporting its occurrence to the appropriate authorities, legal mandates to report suspected such cases are required. Federal child-protection legislation to protect children in the UAE is currently in place and is becoming more familiar. The need for greater education in this field has been expressed even among health professionals with immense levels of engagement with child protection [32]. In Jordan, 124 (32.4%) undergraduates and 23 (39.7%) postgraduates dental students did not know where to report child abuse and neglect [33]. Strong endorsement advocating the need for education regarding all applied ethics topics is not surprising as lack of relevant training has been noted in other countries such as Germany [34] and the USA [35].

Despite the equal curricular exposure to the subject of ethics among male and female students in this cohort, women were recommending more curricular attention to informed consent related topics. This is consonant with other experiences, Bickle and Ruffin [36], reported that women were more likely to claim numerous subjects as inadequately covered by the curriculum than men. These findings are also in congruence with a similar study conducted in North America by Roberts et al, who found evidence to support their hypothesis that female students in training more strongly expressed interest in curricula attention to improvements in bioethics principles, informed consent related topics and special population issues [15]. One reason could be that women tend to give more weight to patient education, community services and psychological aspects of patient care [36] than men do in their choice of a medical career. Accordingly, they could have higher expectations regarding opportunities to master these goals in medical school. The other reason could be that women are receiving less attention and encouragement from faculty members compared with their male peers [36]. As faculty members continue to be more males, they tend to be more comfortable with male than female as suggested by Bickle and Ruffin [36]. Another potential reason is that women tend to underestimate their performances and abilities [37]. A study of a surgery clerkship reported that female students gave less appraisal to 12 of 15 aspects of the clerkship and the overall assessment compared with that of males, even though the performances were comparable between the two genders [38].

Lapid et al [17], reported that residents who reported encountering ethical dilemmas more frequently wanted more education on these topics. In this cohort; although not significant; there was a minor tendency for students who registered lower scores on the item “during your medical training, how often have you been treated in a professional manner by your supervising residents, faculty, and training institution” to have slightly less perception for educational needs for various topics compared with those students who scored higher. One could speculate that these students might have had experiences that resulted in unfavorable impressions toward ethics as a subject. We also found that there was significantly greater endorsement by students who scored low on the item “being treated in professional manner” for the need for education regarding the care of adolescents. This increased interest may reflect a concern to integrate these educational experiences into a personal philosophy of medicine [39]. It may also suggest underlying interest to get involved in learning activities that stimulate particular curiosity, has particular meaning to students and/or for satisfaction derived from it. Faculty can facilitate intrinsic desire by allowing students to engage in learning activities that provide a sense of self-reliance. Studies exploring medical student motivation have shown that intrinsically motivated students persist longer at tasks, have higher levels of meaning orientation and better performance [40,41].

The main limitation of this study is that it assessed students’ perspectives at a one point in time and a sole institute. Hence, a longitudinal, larger scale study is necessary to assess their validity and generalizability. Additionally, the cohort included a cohesive sample with similar ethics learning exposure thus making it difficult to assess changes in the students’ attitudes. Furthermore, the students' knowledge on informed consent items was not measured in this study. The survey was administered in English but participants' fluency in this language was not confirmed as part of the study, however proficiency in English is essential for admission to our medical school at UAE University, requiring a minimal score of five (International Language Testing System, IELTS or an equivalent Test of English as a Foreign Language (TOEFL) score.The study’s power evolves from highlighting student views about ethics and professionalism topics in undergraduate medical education in a region where such studies are scant. This information also may help promote curricular content and training methods that are more acceptable to students as has been previously suggested [42]. Also given the increased educational focus on ethics and professionalism internationally, medical ethics educators need to develop a broad understanding of the factors relevant to the learner and how they are linked to experiences and conflicts detected in clinical rotations.

## Supporting information

S1 File STROBE-checklist. STROBE-checklist.
(PDF)

S2 File Minimal data set. (XLSX)
